# Pathways and Bioenergetics of Anaerobic Carbon Monoxide Fermentation

**DOI:** 10.3389/fmicb.2015.01275

**Published:** 2015-11-19

**Authors:** Martijn Diender, Alfons J. M. Stams, Diana Z. Sousa

**Affiliations:** ^1^Laboratory of Microbiology, Wageningen UniversityWageningen, Netherlands; ^2^Centre of Biological Engineering, University of MinhoBraga, Portugal

**Keywords:** water–gas shift reaction, syngas, carboxydotrophic, hydrogenogenesis, methanogenesis, acetogenesis

## Abstract

Carbon monoxide can act as a substrate for different modes of fermentative anaerobic metabolism. The trait of utilizing CO is spread among a diverse group of microorganisms, including members of bacteria as well as archaea. Over the last decade this metabolism has gained interest due to the potential of converting CO-rich gas, such as synthesis gas, into bio-based products. Three main types of fermentative CO metabolism can be distinguished: hydrogenogenesis, methanogenesis, and acetogenesis, generating hydrogen, methane and acetate, respectively. Here, we review the current knowledge on these three variants of microbial CO metabolism with an emphasis on the potential enzymatic routes and bio-energetics involved.

## Introduction

Carbon monoxide is a tasteless, odorless gas, best known for its toxic characteristics. It is part of the global carbon cycle, is involved in assimilatory and dissimilatory pathways of numerous microorganisms and was found to act as a signal molecule in mammals (Ryter and Otterbein, 2004). Additionally, it is speculated to be an important substrate for early life on earth (Miyakawa et al., 2002). CO is chemically formed during oxygen limited combustion of carbon materials, and can be biologically generated via cellular metabolism (Eikmanns et al., 1985; Ryter and Otterbein, 2004) or enzymatic degradation of heme (Chin and Otterbein, 2009). A large part of naturally generated CO is assumed to be formed via photochemical reactions (Weinstock and Niki, 1972). Other sources contributing to the atmospheric CO level are: volcanic activity, forest fires, and over the last two centuries industrial activity. Cumulative, these activities result in the production of approximately 2.6 petagram (Pg) CO per year (Khalil and Rasmussen, 1990).

Due to the foreseen depletion of fossil energy sources and consideration on environmental impact of current chemical industry, alternative sustainable technologies are being developed. A bio-based economy is considered one of the sustainable solutions for the growing resource depletion, and has potential to replace petroleum industries in the future. However, the hydrolysis of non-food-competing, ligno-cellulosic biomass limits the application of bio-based technologies, leaving a significant amount of the initial substrate unused (Hamelinck et al., 2005). Gasification of the carbohydrate material, forming syngas, is a potential way of gaining access to all material of the original source, mainly in the form of CO, H_2_, and CO_2_. A large spectrum of carbon sources can fuel this technology, from coal, tar, and gas to ligno-cellulosic biomass, all kinds of municipal waste, and digester sludge. As syngas mainly consists of CO, H_2_, and CO_2_, autotrophic, CO-tolerant microorganisms are required as biocatalysts for fermentation of this gas. Numerous microorganisms have shown to utilize CO as a substrate, producing organic compounds such as acetate, ethanol, 2,3-butanediol, and butyrate (Latif et al., 2014; Tiquia-Arashiro, 2014; Dürre and Eikmanns, 2015). In addition, methanogenesis and hydrogenogenesis using syngas as a substrate could have large implications for biofuel production. Even though production of interesting chemicals from syngas is possible, the exact metabolism of syngas conversion is not fully understood. This limits the optimization of potential production strains and, thus, the development of bio-based production processes. Different aspects of biological conversion of CO and syngas have been reviewed in the past decade (Henstra et al., 2007b; Oelgeschlager and Rother, 2008; Bengelsdorf et al., 2013; Sokolova and Lebedinsky, 2013; Latif et al., 2014; Dürre and Eikmanns, 2015). However, a large part of these reviews focus on industrially relevant species and their application potential, while only few address fundamental aspects.

Two types of CO metabolism can be distinguished: respiratory and fermentative (**Table 1**), the former involving an exogenous electron acceptor, whereas the latter utilizes internally generated intermediates as electron acceptor. A relatively well studied example of respiratory CO metabolism is CO oxidation coupled to oxygen reduction (Meyer and Schlegel, 1983; Meyer et al., 1986). Other, less characterized, electron acceptors that have been identified for carboxydotrophic growth are sulfate (Parshina et al., 2005a, 2010), anthroquinone disulfonate (AQDS; Henstra and Stams, 2004), fumarate (Henstra and Stams, 2004), and ferrihydrite (Slobodkin et al., 2006). The main focus of this review is on fermentative CO metabolism, distinguishing hydrogenogenesis, acetogenesis, and methanogenesis with a special emphasis on the potential enzymatic routes and bio-energetics involved. Classifying hydrogenogenesis as a fermentative process is debatable because protons can be considered exogenous acceptors. However, as protons are present in any microbial environment and are generated from the substrate water during hydrogenogenesis, hydrogenogenic metabolism is considered here as fermentative.

**Table 1:** Reaction equations and their standard Gibbs free energy (ΔG^0^) for several modes of carboxydotrophic growth.

Metabolism		Reaction	ΔG^0^ (kJ)
Fermentative			
	Hydrogenogenic	CO + H_2_O → CO_2_ + H_2_	–20
	Methanogenic	4 CO + 2 H_2_O → CH_4_ + 3 CO_2_	–210
	Acetogenic	4 CO + 2 H_2_O → CH_3_COO^-^ + H^+^+ 2 CO_2_	–174
	Solventogenic (ethanol)	6 CO + 3 H_2_O → C_2_H_5_OH + 4 CO_2_	–224
Respiratory			
	Oxygen	2 CO + O_2_ → 2 CO_2_	–514
	Sulfate	4 CO + SO_4_^2-^ + H^+^ → 4 CO_2_ + HS^-^	–231

## General Microbial Co Metabolism

Both aerobic and anaerobic CO metabolism process the CO molecule via the enzyme: carbon monoxide dehydrogenase (CODH). Here, we only consider the anaerobic CODHs, which differ from the aerobic CODHs by structure and the presence of nickel–iron clusters in their active centers (Jeoung et al., 2014). About 6% of all known microbial genomes contain at least one [Ni–Fe] CODH gene sequence, out of which 43% contain at least two, suggesting a more widespread anaerobic CO-utilizing capability than assumed before (Techtmann et al., 2012). It has been shown that CODH genes cluster according to function, instead of clustering by phylogeny, suggesting horizontal gene transfer events have led to the establishment of the CODH gene in the different microbial genomes (Techtmann et al., 2012). CODHs from different organisms have been purified and characterized, including the ones from three relatively well studied anaerobic carboxydotrophic organisms: *Rhodospirillum rubrum* (Drennan et al., 2001), *Carboxydothermus hydrogenoformans* (Dobbek et al., 2001), and *Moorella thermoacetica* (Doukov et al., 2002; Darnault et al., 2003). All the structures of these anaerobic CODHs contain iron-sulfur center (Hu et al., 1996; Dobbek et al., 2001). Additionally, these CODHs contain nickel as a cofactor, for binding and coordinating CO in the active site (Dobbek et al., 2001; Drennan et al., 2001). Other divalent metals were found in the active center of anaerobic CODHs, however, only the nickel containing enzymes were observed to be active in CO conversion (Darnault et al., 2003). CODH can be mono- or bi-functional, both enabling the organism to utilize CO for the energy metabolism. The bifunctional CODH is associated with an acetyl-CoA synthase (ACS), and additionally has a role in carbon fixation, catalyzing the condensation of CO, CoA-SH, and a methyl-group into acetyl-CoA. The bacterial CODH and ACS are connected via a hydrophobic tunnel (Maynard and Lindahl, 1999; Seravalli and Ragsdale, 2000; Lindahl, 2002), preventing CO from being a toxic intermediate in the metabolism of the cell (Doukov et al., 2002).

The redox potential of the CO/CO_2_ pair (*E*^0^ = –520 mV), is lower than that of H_2_/H^+^ (*E*^0^ = –414 mV), which has significant implications for the metabolism. While a metabolism driven by hydrogen requires bifurcation mechanisms to reduce ferredoxin (*E*^0^ = –400 mV) (Buckel and Thauer, 2013), CO can solely drive this reaction. However, the more negative redox potential of CO poses a challenge for the redox balance of the organism. Therefore, efficient cofactor re-oxidizing pathways are required to avoid the cell from becoming completely reduced. Additionally, hydrogenases are considered to be a weak point in CO metabolism, as hydrogen metabolism is often observed to be rapidly inactivated upon CO exposure (Purec et al., 1962; Daniels et al., 1977; Genthner and Bryant, 1982; Bertsch and Müller, 2015). However, [Ni–Fe]-hydrogenases were found to be less sensitive to CO than [Fe–Fe] or iron-only hydrogenases (Adams, 1990b; De Lacey et al., 2007). Some microorganisms possess [Ni–Fe] hydrogenases that are highly tolerant to CO, such as *Rhodospirillum rubrum* (Fox et al., 1996b) and *Pyrococcus furiosus* (Adams, 1990a). CO is known to strongly bind to metals via a process called back bonding, which is also considered to be the mechanism of toxicity (Jeoung et al., 2014).

## Co Driven Hydrogenogenic Metabolism

Coupling CO oxidation to proton reduction is, conceptually seen, one of the simplest mechanisms of biological energy conservation. This reaction, also known as the water–gas shift reaction, results in the formation of hydrogen and CO_2_ (**Table 1**). The reaction was found to be completed by three enzymes: CODH, an electron transfer protein and an energy converting hydrogenase (EcH). CO is oxidized via the CODH complex, and electrons are transferred to a “ferredoxin-like” electron carrier. Oxidation of this electron carrier can be coupled to proton reduction via an EcH complex, producing hydrogen and simultaneously generating an ion motive force (Hedderich and Forzi, 2004). Besides being involved in hydrogenogenic metabolism, EcH enzymes also play a role in sugar fermentation (Sapra et al., 2003) and methanogenesis (Thauer et al., 2010). Several microorganisms which hydrogenogenically metabolize CO have been isolated; most of them are thermophiles (**Table 2**). Two microorganisms conserving energy via the water–gas shift reaction have been rather well studied, the mesophilic *Rhodospirillum rubrum* (Kerby et al., 1995) and the thermophilic *Carboxydothermus hydrogenoformans* (Svetlichny et al., 1991).

**Table 2:** Isolated micro-organisms capable of conserving energy from the water–gas shift reaction.

Species	Origin	Temperature optimum (°C)	Carboxydotrophic generation time (h)	Reference
**Mesophilic bacteria**				
*Rhodospirillum rubrum*	Various environments	30	5 (dark, acetate)	Kerby et al., 1995
*Rubrivivax gelatinosa*	Lake sediment	34	6.7 (dark, trypticase) 10 (light, autotrophically) 1.5 (light, malate)	Uffen, 1976; Maness et al., 2005
*Rhodopseudomonas palustris*	Anaerobic wastewater sludge digester	30	2 (light, autotrophically)	Jung et al., 1999
**Thermophilic bacteria**				
*Caldanaerobacter subterraneus* ssp. *pacificus*	Submarine hot vent, Okinawa Trough	70	7.1	Sokolova et al., 2001; Fardeau et al., 2004
*Carboxydocella sporoproducens*	Hot spring, Karymskoe Lake	60	1	Slepova et al., 2006
*Carboxydocella thermoautotrophica*	Terrestrial hot vent, Kamchatka Peninsula	58	1.1	Sokolova et al., 2002
*Carboxydothermus hydrogenoformans*	Freshwater hydrothermal spring, Kunashir Island	70	2	Svetlichny et al., 1991
*Carboxydothermus islandicus*	Hot spring, Hveragerdi	65	2	Novikov et al., 2011
*Carboxydothermus pertinax*	Volcanic acidic hot spring, Kyushu Island	65	1.5	Yoneda et al., 2012
*Carboxydothermus siderophilus*	Hot spring, Kamchatka Peninsula	65	9.3	Slepova et al., 2009
*Dictyoglomus carboxydivorans*	Hot spring, Kamchatka Peninsula	75	60	Kochetkova et al., 2011
*Moorella stamsii*	Digester sludge	65	N.D.	Alves et al., 2013
*Thermincola carboxydiphila*	Hot spring, Lake Baikal	55	1.3	Sokolova et al., 2005
*Thermincola ferriacetica*	Hydrothermal spring, Kunashir Island	60	N.D.	Zavarzina et al., 2007
*Thermincola potens*	Thermophilic microbial fuel cell	55	N.D.	Byrne-Bailey et al., 2010
*Thermolithobacter carboxydivorans*	Mud and water, Calcite Spring	73	1.3	Sokolova et al., 2007
*Thermosinus carboxydivorans*	Hot spring, Norris Basin	60	1.15	Sokolova et al., 2004a
*Thermoanaerobacter thermohydrosulfuricus* ssp. *carboxydovorans*	Geothermal spring, Turkey	70	N.D.	Balk et al., 2009
*Desulfotomaculum carboxydivorans*	Paper mill wastewater sludge	55	N.D.	Parshina et al., 2005b
**Thermophilic archaea**				
*Thermococcus onnurineus*	Deep-sea hydrothermal vent	80	5	Bae et al., 2006, 2012
*Thermocuccus AM4*	Hydrothermal vent	82	5	Sokolova et al., 2004b
*Thermofilum carboxyditrophus*	Kamchatka hot springs	90	N.D.	Kochetkova et al., 2011

*Not determined parameters are marked N.D.*

*Rhodospirillum rubrum*, *Rubrivivax gelatinosa*, and *Rhodopseudomonas palustris* are photosynthetic bacteria and the only known mesophiles capable of efficiently conserving energy from the water–gas shift reaction (**Table 2**). In *R. rubrum* the CO-dependent metabolism is regulated via a heme-protein, which acts as CO sensor (CooA) and controls transcription of the enzymatic machinery required for CO dependent growth (Roberts et al., 2001). The genes controlled by CooA in *R. rubrum* are arranged in two gene clusters: *cooFSCTJ* and *cooMKLXUH*. The first gene cluster codes for the active CODH (*cooS*), electron carrier (*cooF*) and a nickel inserting complex (*cooCTJ*) (Kerby et al., 1997), whereas the latter codes for a six subunit EcH complex. The CODH structure of *R. rubrum* has been resolved to 2.8Å, and is similar to the CODH of anaerobes such as *C. hydrogenoformans* and *M. thermoacetica* (Dobbek et al., 2001; Drennan et al., 2001). Electrons from CO oxidation are transferred to an iron–sulfur protein (CooF), which shuttles the electrons to the EcH complex. The CooF complex is tightly associated with the CODH, and was shown to be reduced upon CO exposure (Ensign and Ludden, 1991). Other electron carriers, such as other native ferredoxins from *R. rubrum*, were ineffective in mediating electron transfer from CODH to the hydrogenase (Ensign and Ludden, 1991). This suggests that the CooF subunit is highly specific for electron transfer from CODH to the hydrogenase. Not only is the *R. rubrum* CODH efficient in converting CO to CO_2_, also its CO-induced hydrogenase is well adapted to CO dependent growth (Bonam et al., 1989). The EcH of *R. rubrum* consists of 6 subunits, of which two subunits, CooH and CooL, are similar to the ones found in some [Ni–Fe] hydrogenases. In addition, all six subunits show high similarity with complex I NADH:oxidoreductases, which are involved in proton translocation coupled to NADH oxidation (Fox et al., 1996a,b). During activity assays, the EcH is found to function optimally in presence of CODH:CooF, which is theorized to promote forming and maintaining a stable complex (Singer et al., 2006). The CO-induced hydrogenase of *R. rubrum* is highly CO tolerant, and only shows signs of inhibition above 60% CO in the headspace (Fox et al., 1996b). Despite the seemingly efficient water–gas shift metabolism in *R. rubrum*, autotrophic growth on solely CO as a carbon source is very slow (Dashekvicz and Uffen, 1979). *R. rubrum* requires small amounts of yeast extract and acetate as a carbon source to grow efficiently. *R. gelatinosus* and *R. palustris* exhibit a similar hydrogenogenic CO metabolism as *R. rubrum*. However, in contrast to *R. rubrum*, these bacteria were able to perform the water–gas shift reaction and grow on CO as a sole carbon source, but merely in presence of light (Jung et al., 1999; Maness et al., 2005). Growth was significantly slowed down for *R. gelatinosus* in the dark, which was not assessed for *R. palustris*. The growth rate of *R. gelatinosus* increased significantly after addition of malate as a carbon source (Maness et al., 2005). So, despite efficient energy conservation via the water–gas shift reaction, as shown in the presence of organic carbon sources, autotrophic growth seems very energy intensive for these phototrophic bacteria. The relatively slow growth on CO as a sole carbon source is likely due to the use of the energy demanding Calvin-cycle, which can be considered the main carbon-fixation mechanism in these phototrophic bacteria. All three isolated mesophilic, hydrogenogenic carboxydotrophs are phototrophs. However, it is unclear why the trait of hydrogenogenic CO-utilization among mesophiles is exclusive to this group.

Among thermophilic hydrogenogens, *C. hydrogenoformans* is one of the best studied. This bacterium was first thought to only grow fermentatively on CO or pyruvate as substrate, but was later shown to be capable of respiratory growth with CO as well (Henstra and Stams, 2004). With five different CODHs encoded in its genome, it is one of the few organisms known to have multiple CODH types, which is likely related to its exceptional growth capabilities on CO (Wu et al., 2005). *C. hydrogenoformans* uses a CODH–CooF–EcH complex, which is highly similar to the system found in *R. rubrum* (Soboh et al., 2002). In contrast to the mentioned mesophilic phototrophs, *C. hydrogenoformans* is capable of efficient autotrophic growth, using solely CO as energy and carbon source. This characteristic might be assigned to the presence of the Wood–Ljungdahl pathway, which in contrast to the Calvin-cycle is not as energy demanding. Additionally, a turnover rate of 31000 s^-1^ was found for the CODHII of *C. hydrogenoformans* (Svetlitchnyi et al., 2001), allowing for fast generation of reduction equivalents and thus a quick energy metabolism. Upon increased hydrogen and carbon dioxide pressure, acetate is produced from CO by *C. hydrogenoformans*. This suggests acetogenic use of the Wood–Ljungdahl pathway could act as a backup for its hydrogenogenic metabolism (Henstra and Stams, 2011). *C. hydrogenoformans* and related thermophilic species are suggested to fulfill an important role in the volcanic environments they originate from, ensuring CO concentrations are kept below toxic levels, making life of other non-CO tolerant organisms possible (Techtmann et al., 2009). Furthermore, it seems horizontal gene transfer events have played an important role in the establishment of CO-driven hydrogenogenic metabolism in these environments (Techtmann et al., 2012; Sant’Anna et al., 2015).

**FIGURE 1:**
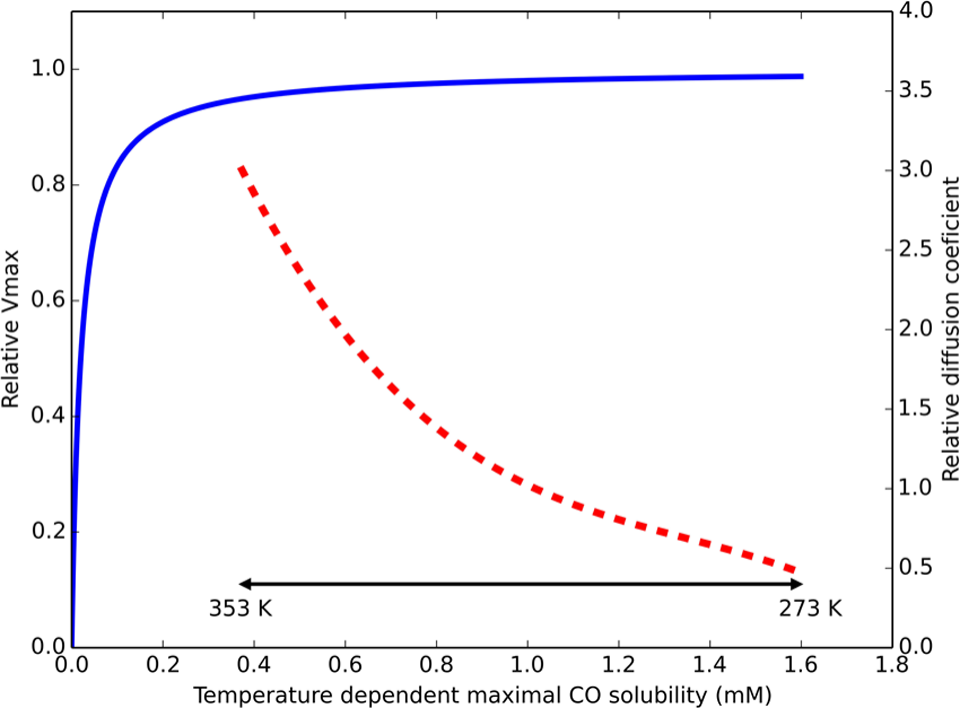
**Temperature effect on parameters for CO driven hydrogenogenic growth**. Estimated relative *V*max of a CO dehydrogenase (CODH), displayed by the blue continuous line, assuming Michaelis–Menten kinetics (Km = 0.03 mM) based on maximal solubilized CO. The approximated ratio of the diffusion constant for any dissolved gas (at atmospheric pressure) in water compared to its diffusion constant at 298 K is displayed by the red dashed line. The physiologically relevant temperature range is displayed by the double headed black arrow. The displayed temperature scale is not linear due to the non-linear relation between temperature and CO solubility.

When assessing the distribution of isolated hydrogenogenic carboxydotrophic microorganisms (**Table 2**), thermophilic isolates seem to be more prevalent than mesophilic ones, which contrasts with the solubility of gaseous substrates at elevated temperatures. Temperature increase has two effects on dissolved gases: decreased gas solubility and increased gas diffusion rates. In a hydrogenogenic metabolism the microorganisms use a gaseous substrate, subsequently producing a gaseous product. The thermodynamics of this metabolism thus relies on the concentration of two gases, which is indirectly related to the diffusion rate of these gases. The Km values of the CODH in *R. rubrum*, *C. hydrogenoformans*, and the acetogen *M. thermoacetica*, are in the order of 0.032, 0.018, and 0.01 mM, respectively (Raybuck et al., 1988; Jeon et al., 2005; Seravalli and Ragsdale, 2008). The maximal solubility of CO in water is approximately 1.6 to 0.38 mM, in the range of 273 to 353 K, respectively. Assuming a Km of 0.03 mM and applying simple Michaelis–Menten kinetics, the associated CODH reaction rate at these CO concentrations goes from 98 to 93% of Vmax (**Figure 1**). This suggests that carboxydotrophic microorganisms are not significantly limited by the maximal solubility of CO at elevated temperatures. The CO diffusion coefficient, an indication of the diffusion rate of the gas, is 2.0 × 10^-5^ cm^2^/s in water at 298 K. Compared to 298 K, the estimated diffusion constant at 333 K is two times larger and three times larger at 353 K (approximated by the Stokes–Einstein equation, using the dynamic viscosity of water, **Figure 1**). This suggests that at increased temperatures, CO is more rapidly supplied to the microorganisms. Via the same mechanism, the temperature indirectly affects the degree of accumulation of hydrogen in the near vicinity of the microorganism, allowing two times faster removal of H_2_ at 333 K, and three times at 353 K. This suggests thermophilic hydrogenogenic metabolism suffers less from hydrogen accumulation to thermodynamically unfavorable levels when compared to mesophilic conditions. Therefore, we hypothesize that CO driven hydrogenogenic metabolism is more favorable at higher temperatures when compared to lower temperatures, giving rise to the currently observed temperature distribution of carboxydotrophic hydrogenogenic isolates (**Table 2**). If this potential advantage also translates into an increased energy yield, and thus a higher growth rate with increasing temperatures is unclear as numerous factors influence the growth rate.

## Co Driven Acetogenesis and Related Metabolisms

Formation of acetate from CO was first reported by Lynd et al. (1982). Since then many acetogens have been discovered to utilize CO, both homo-acetogenic organisms (i.e., generating solely acetate) and mixed-product acetogenic organisms (i.e., generating a mixture of end-products; **Table 3**). Large differences can be observed between the growth rates and yields of CO-grown acetogens, indicating differences in their ways of energy conservation and pathways utilized (**Table 3**). The Wood–Ljungdahl pathway is the central metabolism for acetogenic growth (Ragsdale and Pierce, 2008). In contrast to the Calvin-cycle, the reductive TCA-cycle or the 3-hydroxypropionate cycle, the Wood–Ljungdahl pathway can be used to conserve energy. The Wood–Ljungdahl pathway consists of two branches, which in total require eight reduction equivalents and one ATP to form acetyl-CoA from two CO_2_. During formation of acetate the ATP investment is regained by substrate level phosphorylation, but no net ATP is gained, thus requiring an ion motive force to conserve net energy. Complex I related RnF complexes have been identified as the cation extruding mechanism in many acetogens, linking ferredoxin oxidation to NAD reduction, simultaneously forming either a sodium or proton gradient (Biegel et al., 2011). Upon consumption of this gradient, ATP is formed from ADP and phosphate via an ATPase (Müller, 2003). The availability of ferredoxin can thus be considered the driving force of energy generation in the autotrophic acetogenic metabolism. It is assumed that in hydrogenotrophic acetogens electron-bifurcating hydrogenases are required to generate reduced ferredoxin (Poehlein et al., 2012; Buckel and Thauer, 2013). As CO can directly reduce ferredoxin, these bifurcating systems seem redundant during CO-driven growth, but might be utilized to correctly distribute reduction equivalents over the metabolism. Four steps in the Wood–Ljungdahl pathway require input of electrons for fixation of two CO_2_. The type of cofactors utilized in each of these steps differs per enzyme and per microorganism, making it impossible to propose a general metabolism for acetogenic CO metabolism. This distinct use of cofactors can explain part of the differences observed in yield and growth rate of different acetogens (**Table 3**). Recently, the energy metabolism for autotrophic growth on H_2_/CO_2_ for *Acetobacterium woodii*, *M. thermoacetica*, and *Clostridium ljungdahlii* was reviewed (Schuchmann and Müller, 2014). Here, we assess and compare the CO metabolism of these three species, which are the current model organisms for acetogenic metabolism.

**Table 3:** Properties of CO grown acetogenic microorganisms.

Organism	Energy conservation: mechanism (cation)	Minimal generation time reported, (h)		Biomass yield (gCells/mol)		Reference
				
		CO	H_2_	CO	H_2_	
**Mixed fermenters**						
*Clostridium ljungdahlii*	RnF (H^+^)	12^A^ (DM) 3.8 (UM)	22^A^ (DM)	1.38 (DM) 8.4^C^ (UM)	0.37 (DM)	Tanner et al., 1993; Phillips et al., 1994; Younesi et al., 2005; Cotter et al., 2009; Köpke et al., 2011
*Clostridium autoethanogenum*	RnF (H^+^)	4 (UM)	N.D.	N.D.	N.D.	Abrini et al., 1994; Cotter et al., 2009; Köpke et al., 2011
*Clostridium formicoaceticum*	RnF (N.D.)	7 (UM)	No growth	N.D.	N.A.	Lux and Drake, 1992
*Clostridium ragsdalei*	RnF (N.D.)	4 (UM)	N.D.	N.D.	N.D.	Köpke et al., 2011; Kundiyana et al., 2011
*Clostridium scatologenes*	RnF (N.D.)	11.6 (UM)	17.3 (UM)	N.D.	N.D.	Liou et al., 2005
*Clostridium drakei*	RnF (N.D.)	5.8 (UM)	3.5 (UM)	N.D.	N.D.	Küsel et al., 2000; Liou et al., 2005
*Clostridium carboxidivorans*	RnF (H^+^)	4.3 (UM)	5.8 (UM)	0.25	N.D.	Rajagopalan et al., 2002; Liou et al., 2005
*Alkalibaculum bacchi*	RnF (N.D.)	5.8 (UM)	N.D.	0.8–2 (UM)	N.D.	Allen et al., 2010; Liu et al., 2012
*Butyribacterium methylotrophicum*	RnF (N.D.)	13.9^A^ (UM)	9 (UM)	3 (UM)	1.7 (UM)	Lynd et al., 1982; Lynd and Zeikus, 1983; Heiskanen et al., 2007
*Eubacterium limosum*	RnF (Na^+^)	7 (UM)	∼20^B^ (UM)	3.38 (UM)	0.84 (UM)	Genthner and Bryant, 1982, 1987; Jeong et al., 2015
*Oxobacter pfennigii*	RnF (N.D.)	13.9^A^ (UM)	No growth	2.50 (UM)	N.A.	Krumholz and Bryant, 1985
**Homo-acetogenic**						
*Moorella thermoautotrophica*	EcH/ Cytochromes (H^+^)	7 (UM), 9 (DM)	33 (DM)	3.34 (UM) 2.53 (DM)	0.82 (DM)	Savage and Drake, 1986; Savage et al., 1987; Collins et al., 1994
*Moorella thermoacetica*	EcH/ Cytochromes (H^+^)	9 (DM)	16 (DM)	1.28 (UM)	0.46 (UM)	Daniel et al., 1990; Collins et al., 1994; Pierce et al., 2008
*Acetobacterium Woodii*	RnF (Na^+^)	13^D^ (UM), 5.5^A^ (UM ^+^ formate)	6.2^A^ (UM)	N.D.	1.05^C^ (DM)	Balch et al., 1977; Tschech and Pfennig, 1984; Genthner and Bryant, 1987; Hess et al., 2013; Bertsch and Müller, 2015
*Blautia producta*	RnF (Na^+^)	1.5 (UM)	5 (UM)	2.13^C^ (UM)	0.65^C^ (UM)	Lorowitz and Bryant, 1984; Geerligs et al., 1989
*Clostridium aceticum*	RnF (N.D.)	∼10^B^ (UM)	20 (UM)	N.D.	N.D.	Braun et al., 1981; Sim et al., 2007
**Acetogenic Archaea**						
*Archeoglobus fulgidus*	SLP	∼10	N.A.	N.D.	N.A.	Henstra et al., 2007a
*Methanosarcina acetivorans*	SLP, RnF (Na^+^)	∼20	N.A.	2.5	N.A.	Rother and Metcalf, 2004

*Parameters measured in medium containing complex additives like yeast extract or rumen fluid are indicated as undefined medium (UM). Parameters measured in medium of which the exact composition is known are indicated as defined medium (DM). SLP, substrate level phosphorylation. Not determined parameters are marked N.D, whereas not applicable parameters are indicated N.A.*

*^A^Duplication time is recalculated from a given specific growth rate (μ) assuming: 2 = e^μt^.*

*^B^Duplication time is estimated from a given graph, as numbers were not mentioned.*

*^C^Yield was recalculated from g protein/mol CO to gCells/mol CO, making the assumption proteins makes up 60% of the dry weight (Krumholz and Bryant, 1985).*

*^D^*Acetobacterium woodii* was shown to not grow on CO as a sole carbon source; generation time displayed was measured in rich medium likely containing substrates for co-fermentation.*

**FIGURE 2:**
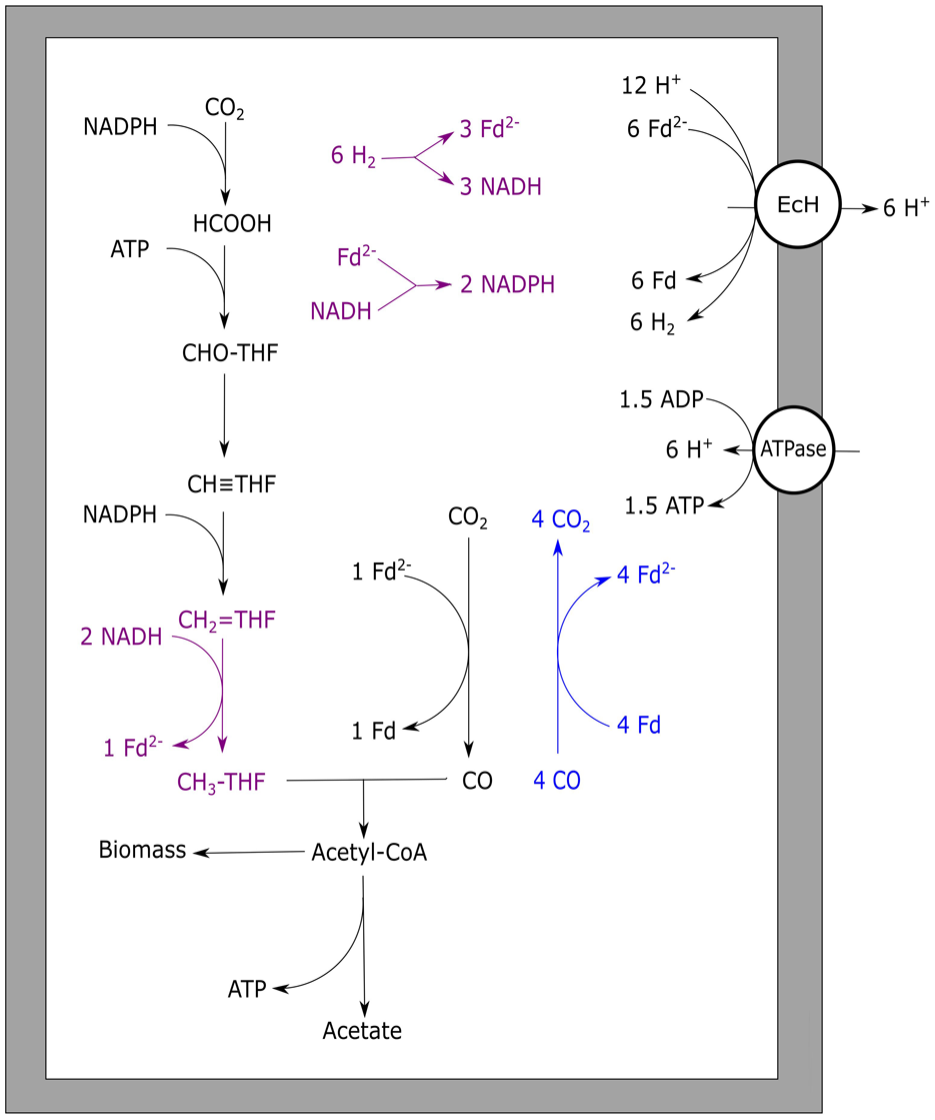
**Carbon monoxide metabolism of *Moorella thermoacetica***. Stoichiometric conversion of CO to acetate by *M. thermoacetica* is disaplayed. Reactions marked blue indicate CO oxidizing activity by CODH, bifurcating reactions are marked purple. The EcH is assumed to transport one proton per hydrogen formed whereas the ATPase is assumed to generate one ATP per four protons translocated. EcH, energy converting hydrogenase; ATPase, ATP synthase; Fd, ferredoxin; THF, tetrahydrofolate.

*Moorella thermoacetica* is one of the best studied homo-acetogenic bacteria able to utilize CO. This organism differs from other acetogenic strains in the sense that it does not possess an RnF complex, leaving the mechanism of cation transport unknown (Pierce et al., 2008). Either an EcH complex or cytochromes are proposed to perform the build-up of an ion motive force. Several cytochromes have been found in *Moorella* species that are potentially active in proton transport (Gottwald et al., 1975). However, the role of these electron carrier proteins in acetogenic metabolism has never been experimentally shown. Additionally, a role of these cytochromes in respiratory metabolism is likely to exist, as growth with nitrate and nitrite was shown to be possible (Drake and Daniel, 2004). An EcH complex is coded for in the genome of *M. thermoacetica* (Huang et al., 2012), making cation export via this enzyme a possibility. Based on presence of an EcH complex, an energy metabolism for growth on H_2_/CO_2_ has been proposed for *M. thermoacetica* (Schuchmann and Müller, 2014). The plausible metabolism is based on the assumption that methylenetetrahydrofolate reductase, responsible for the exergonic reduction of methylenetetrahydrofolate to methyltetrahydrofolate, is somehow coupled to energy conservation. As all enzymes of the Wood–Ljungdahl pathway are considered soluble, none of these is expected to be involved in generation of an ion motive force. Crude membrane extraction methods were thought to be the cause of finding all the enzymes in the soluble fraction, and more gentle extraction methods confirmed membrane attachment of methylenetetrahydrofolate reductase (Hugenholtz and Ljungdahl, 1989). Also in the acetogen *Blautia producta*, methylenetetrahydrofolate reductase was found to be loosely attached to the cellular membrane, supporting a potential role in energy conservation (Wohlfarth et al., 1990). Further experimental evidence for a direct role of this enzyme in energy conservation has never been found though. A recent theory is that the methylenetetrahydrofolate reductase has bifurcation activity, coupling the oxidation of two NADH molecules to the reduction of ferredoxin and methylenetetrahydrofolate (Huang et al., 2012). The hypothesis for a bifurcating function of this enzyme in *M. thermoacetica* is supported by the following arguments: i) the enzyme was found to be associated with subunits similar to F420 non-reducing hydrogenases, also found in archaea for bifurcational reduction of CoM–CoB (Huang et al., 2012), ii) in several acetogens the complex was found to contain flavin (Clark and Ljungdahl, 1984; Wohlfarth et al., 1990), which is thought to be essential for bifurcation of the electrons over the two acceptors (Herrmann et al., 2008; Thauer et al., 2008; Kaster et al., 2011; Nitschke and Russell, 2012; Buckel and Thauer, 2013). The flavin is theorized to donate one electron to a high potential acceptor, leaving the flavin at a “red hot” flavosemiquinone state, capable of reducing a low potential acceptor (Buckel and Thauer, 2013). This can be repeated for another two electrons, obtaining two fully reduced products. In case of methylenetetrahydrofolate reductase, the high potential acceptor would be methylenetetrahydrofolate (*E*^0^ = –117 mV) whereas ferredoxin (*E*^0^ = –400 mV) would be the low potential acceptor. Assuming a bifurcating function of methylenetetrahydrofolate reductase and a proton translocation ratio of 1:1 per hydrogen formed by the EcH complex, the energy yield of H_2_/CO_2_ grown *M. thermoacetica* was suggested to be 0.5 ATP per acetate formed (Schuchmann and Müller, 2014). Applying a model using similar assumptions, the metabolism on CO is expected to yield 1.5 ATP per acetate formed (**Figure 2**). The suggested three times increase in energy yield matches with the increased observed growth yield of *M. thermoacetica* with CO (**Table 3**). The same yield increase is observed in the related organism *M. thermoautotrophica*, which is thought to exhibit a similar metabolism (**Table 3**).

**FIGURE 3:**
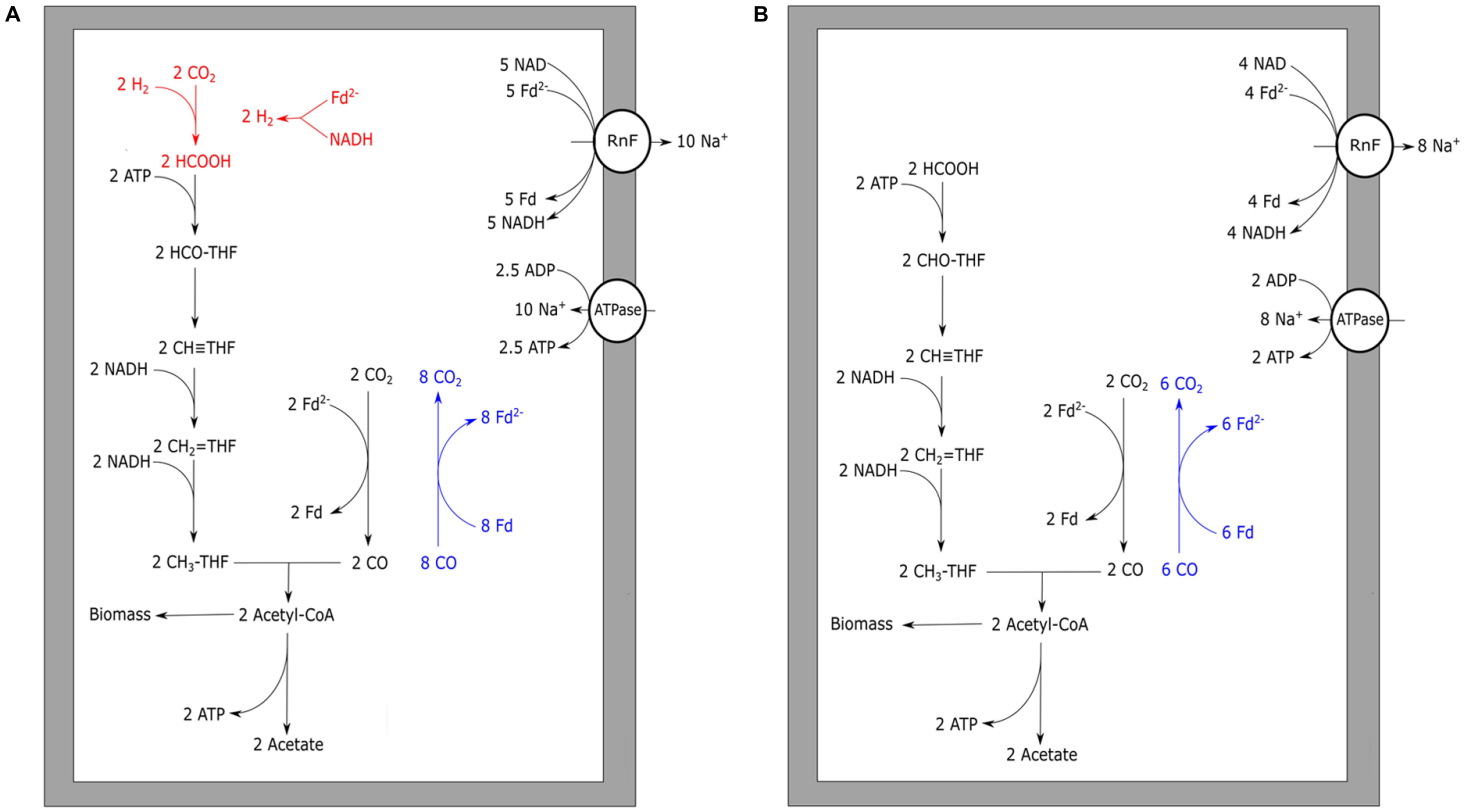
**Carbon monoxide metabolism of *Acetobacterium woodii***. Acetogenic CO metabolism of *A. woodii* is displayed. **(A)** The proposed theoretical pathway and energetic yield of CO conversion to acetate. Pathways prone to CO inhibition are shown in red. **(B)** Proposed metabolism and energetic yield of acetogenic metabolism driven by CO co-fermented with formate. Reactions marked blue indicate CO oxidizing activity by CODH. The RnF complex is assumed to transport two sodium ions per Fd oxidized whereas the ATPase is assumed to generate one ATP per four sodium ion translocated. Rnf, RnF complex; ATPase, ATP synthase; Fd, ferredoxin; THF, tetrahydrofolate.

Initially, the acetogen *Acetobacterium woodii* was reported to grow homo-acetogenically on CO as a sole energy source (Genthner and Bryant, 1987). However, recently it was shown that the organism can only utilize CO in co-fermentation with either hydrogen or formate (Bertsch and Müller, 2015). Additionally, *A. woodii* was shown to produce ethanol when the pressure of CO in the headspace was over 25 kPa (Bertsch and Müller, 2015). These contradictory observations might be explained by the rich undefined medium (UM) used in the initial study, which makes it possible that the organism has co-fermented CO with other substrates, such as formate (Bertsch and Müller, 2015). This is further supported by findings of the initial study that it was not possible to grow *A. woodii* with solely CO on defined medium (Genthner and Bryant, 1987). For *A. woodii*, a metabolism on H_2_/CO_2_ was proposed based on genomic data (Poehlein et al., 2012), and was later adapted suggesting a gain of ∼0.3 ATP per acetate formed (Schuchmann and Müller, 2014). *A. woodii* is suggested to contain a non-bifurcating methylenetetrahydrofolate reductase, using only NADH to form methyltetrahydrofolate (Schuchmann and Müller, 2014). Growth on H_2_/CO_2_ for *A. woodii* was reported with a generation time of approximately 6 h. Presence of increasing levels of CO negatively affected the growth rates on H_2_/CO_2_, and became almost fully inhibited above 15 kPa CO (Bertsch and Müller, 2015). *A. woodii* efficiently co-fermented CO (25 kPa) with formate at a generation time of approximately 5.5 h. Levels up to 50 kPa CO stimulated growth when co-fermenting formate, however, higher CO pressures caused a decrease in growth rate. Growth remained possible up to a maximally tested pressure of 100 kPa CO (Bertsch and Müller, 2015). The inhibitory effect of CO on the *A. woodii* metabolism is suggested to be related to its formate dehydrogenase (Bertsch and Müller, 2015), which is associated with a [Fe–Fe] hydrogenase (Schuchmann and Müller, 2013). Additionally, bifurcational [Fe–Fe] hydrogenases present in *A. woodii* can be a bottleneck in utilization of CO (**Figure 3A**). In the presence of formate, CO-inhibited enzymes are expected not to be required, facilitating the use of CO as a substrate (**Figure 3B**).

**FIGURE 4:**
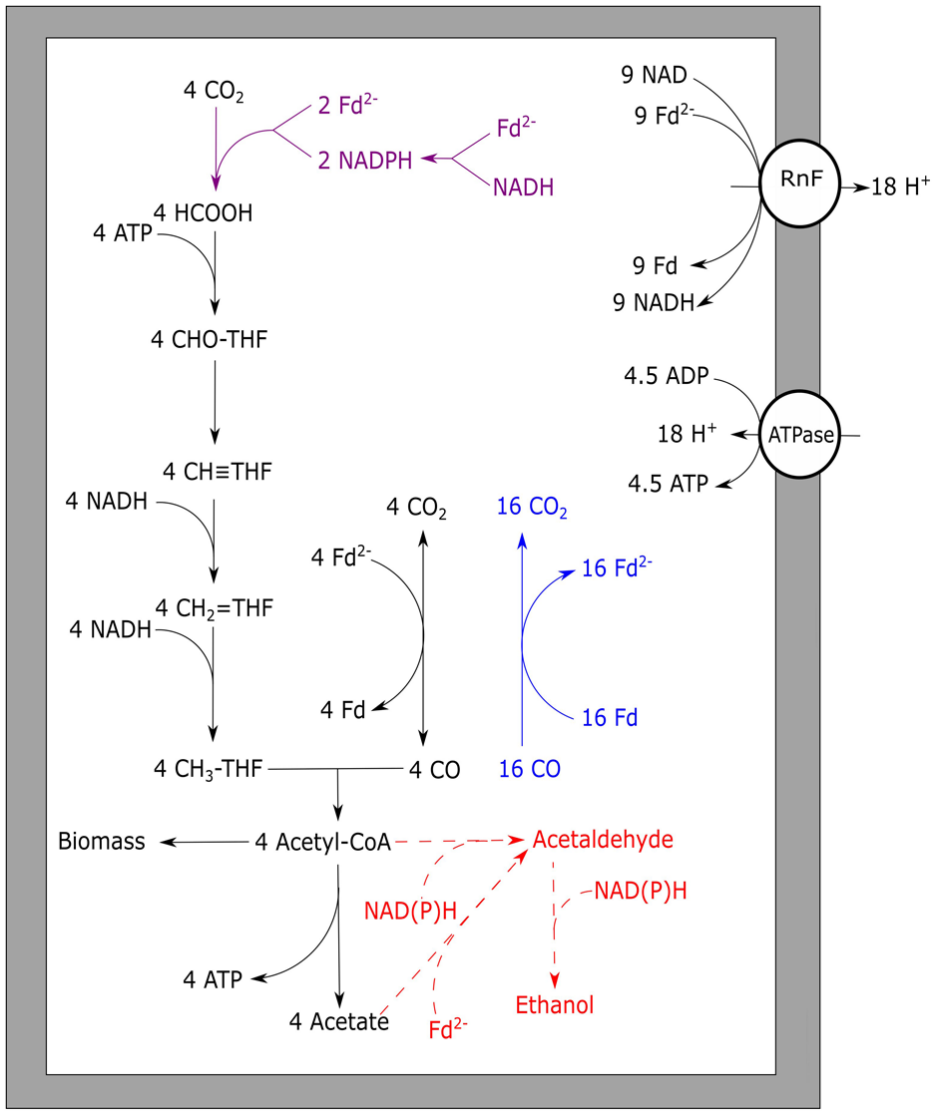
**Carbon monoxide metabolism of *Clostridium ljungdahlii***. Stoichiometric conversion of CO to acetate for *C. ljungdahlii* is displayed. The pathways of ethanol formation are indicated by the dotted line in red, and are not taken into account for the energy yield displayed. Reactions marked blue indicate CO oxidizing activity by CODH, bifurcating reactions are marked purple. The RnF complex is assumed to transport two protons per Fd oxidized whereas the ATPase is assumed to generate one ATP per four protons translocated. Rnf, RnF complex; ATPase, ATP synthase; Fd, ferredoxin; THF, tetrahydrofolate.

Unlike *M. thermoacetica*, which contains an EcH, RnF-utilizing acetogens cannot directly couple ferredoxin oxidation to proton reduction. Therefore, to prevent a completely reduced state of the cell, RnF-containing acetogens need to stoichiometrically couple CO oxidation to the Wood–Ljungdahl pathway. When compared to hydrogenotrophic acetogenesis, acetogenic growth on CO often leads to the formation of additional alcohols, hydrogen, or fatty acids. These by-products are likely formed due to the more strongly reduced environment created by CO. An example is the CO metabolism of *E. limosum*, which produced solely acetate when grown on H_2_/CO_2_, but generates a mixture of acetate and butyrate when grown on CO (Jeong et al., 2015). The best studied pathway for maintaining redox balance during acetogenic growth on CO is solventogenesis. The model organism for this type of fermentation is *C. ljungdahlii*, which is known for its fast growth rate and solventogenic production characteristics on CO (Köpke et al., 2010). In contrast to *A. woodii* and *M. thermoacetica*, the enzymes from the Wood–Ljungdahl pathway in *C. ljungdahlii* have not been purified and tested for cofactor specificity. Therefore, the proposed metabolism of *C. ljungdahlii* on H_2_/CO_2_ was partly based on genomic data (Schuchmann and Müller, 2014). The proposed metabolism on H_2_ is to yield a minimum of 0.13 ATP per acetate formed, and can go up to 0.63 ATP per acetate, depending on which cofactors are utilized in each of the steps. Based on the assumptions of the minimal H_2_ metabolism, a model for CO driven acetogenic growth can be proposed for *C. ljungdahlii*, and related bacteria (**Figure 4**). The model shows a yield of about 1.125 ATP per acetate formed (**Figure 4**), which is further reduced when formation of side products is taken into account. Despite the fact that the energy yield per acetate formed is less as proposed for *M. thermoacetica*, the generation time of *C. ljungdahlii* is shorter. Two mechanisms might contribute to this enhanced growth rate: a bifurcational formate dehydrogenase, and the up-regulation of re-oxidizing reactions. A related bacterium, *Clostridium autoethanogenum*, was found to highly express a formate dehydrogenase associated with a [Fe–Fe]-bifurcating hydrogenase. This formate dehydrogenase is suggested to use one mole of NADPH and one mole of ferredoxin to reduce two moles of CO_2_ to formate (Wang et al., 2013). Despite the sensitivity of [Fe–Fe]-hydrogenases to CO, levels in the cell are assumed to be kept low enough for the hydrogenase to function. The utilization of both NADPH and ferredoxin results in re-oxidation of these two important cofactors, and prevents over-reduction of the cell. However, loss of ferredoxin in re-oxidizing reactions reduces its capacity to act as a driving force for cation export via the RnF complex, lowering the energy yield of the overall metabolism. *C. ljungdahlii* codes for a similar formate dehydrogenase complex in its genome, and might thus utilize a similar system during growth on CO. Additionally, solventogenic reactions play a role in maintaining redox balance. Ethanol production in *C. ljungdahlii* occurs via two pathways: a direct or an indirect pathway (Köpke et al., 2010, 2011). The direct pathway forms ethanol via acetaldehyde directly from acetyl-CoA, utilizing an acetaldehyde/alcohol dehydrogenase complex. This pathway is expected to largely reduce the overall energy yield as no ATP is generated via acetate formation. The indirect pathway does not omit the ADP phosphorylation step, maintaining the energy conservation via acetate synthesis. Acetate is theorized to subsequently be reduced with ferredoxin to acetaldehyde via an aldehyde oxidoreductase. This enzyme was shown to be expressed in CO grown *C. ljungdahlii*, and its expression is stimulated by the addition of external acids (Xie et al., 2015). Ethanol is subsequently formed from acetaldehyde via an alcohol dehydrogenase, utilizing additional reducing equivalents such as NADH or NADPH (Köpke et al., 2010). Energy conservation linked to ethanol formation from H_2_/CO_2_ in *C. autoethanogenum* is also expected to run via this indirect pathway (Mock et al., 2015). Additionally, a similar pathway could be responsible for conversion of different carboxylic acids into alcohols, as observed in mixed cultures exposed to syngas (Liu et al., 2014).

As the suggested energy yield per acetate formed from CO for *C. ljungdahlii* is 1.125 ATP (**Figure 4**), 0.125 ATP is generated up to acetyl-CoA formation. This suggests net energy can be yielded by this organism from any product formed from acetyl-CoA which does not require further investment of ATP. This could include compounds such as C4-carboxylic acids, lactate, fatty acids, and a variety of alcohols, which are considered interesting end products of bio-based processes (Dürre and Eikmanns, 2015).

## Carboxydotrophic Methanogenic Metabolism

Methane can be formed anaerobically from different substrates, such as H_2_/CO_2_, formate, methanol, acetate, or methylamines. The physiology and bioenergetics of different types of methanogenic metabolism have been reviewed before (Thauer et al., 2008). CO is a less studied substrate of methanogenesis, and was first reported by Fischer et al. (1931). It has thereafter been shown to be utilized in the metabolism of several methanogenic strains (**Table 4**). Methanogenic growth on CO as sole substrate appears not to be very efficient, as only three species have been reported to grow while producing methane: *Methanobacterium thermoautotrophicus, Methanosarcina acetivorans*, and *Methanosarcina barkeri*. To gain insight in CO utilization by different groups of methanogens, two main types of methanogenic metabolism are of interest: hydrogenotrophic and aceticlastic methanogenesis. CO is an intermediate in both types of methanogenic metabolism: playing a role in anabolism or catabolism of acetyl-CoA. It is therefore not surprising that genes coding for CODH in methanogens are mainly located in the genome as neighbor of an ACS (Techtmann et al., 2012). Some methanogens do, however, possess more than one CODH, which are not all associated with an ACS complex. Examples are *Methanothermobacter marburgensis* and *Methanococcus jannaschii. M. jannaschii* has a CODH which is in located the same operon as a hydrogenase, suggesting that hydrogenogenic carboxydotrophic metabolism is possible. However, many methanogenic strains have never been tested for utilization of CO as a substrate, and their growth potential on CO is therefore unknown. Unlike in many bacteria, the routes for electron transfer from CODH to the rest of the metabolism are not well established for archaea. In general, “ferredoxin-like” proteins are proposed as the acceptor molecules for CODH complexes. This is confirmed for some methanogens, such as *Methanosarcina thermophila* (Terlesky and Ferry, 1988; Abbanat and Ferry, 1991) and *Methanosarcina barkeri* (Fischer and Thauer, 1990), which require ferredoxin to perform CO-dependent reactions. However, cell-free extract of *M. thermoautotrophicus* exhibited F420-reducing activity in presence of CO, while ferredoxin of *Clostridium pasteurianum* was not reduced (Daniels et al., 1977). F420 was also observed to be reduced by purified CODH from *Methanosaeta concilii*, indicating potential ability of this enzyme to reduce this cofactor (Jetten et al., 1989). However, ferredoxin was not tested as acceptor for the CODH complex of *M. concilii*, and thus cannot be excluded as acceptor. When assuming *E* = –500 mV for ferredoxin under physiological conditions (Buckel and Thauer, 2013), it can be expected that ferredoxin is an ideal acceptor for electrons from CO (*E*^0^ = –520 mV). Transfer of electrons to F420 (*E*^0^ = –380 mV) would result in additional energy loss, and would require bifurcation processes to generate reduced ferredoxin. Ferredoxins have in general been shown to be interchangeable over long phylogenetic ranges, as indicated by the similar characteristics of plant and bacterial ferredoxins (Tagawa and Arnon, 1968). However, ferredoxin from spinach did not form a complex with the CODH of *M. thermoacetica* and was not reduced by this bacterial CODH (Drake et al., 1980; Shanmugasundaram and Wood, 1992). Additionally, the observation that the CODH from *R. rubrum* could only effectively donate electrons to the tightly associated CooF complex (Ensign and Ludden, 1991), suggests not all ferredoxins are efficient in receiving in electrons from a CODH complex. Therefore, the observation that some archaeal CODHs do not transfer electrons to non-native ferredoxin might be related to a difference in characteristics, such as structure and location of the special nickel–iron–sulfur clusters present in the CODH and the possibly different mid-point redox potentials of the ferredoxin.

**Table 4:** Methanogenic archaea capable of metabolizing CO.

Species	Native physiology	Experimental procedure used	Inhibitory levels^A^	Products from CO	Generation time on CO (h)	Reference
**Mesophilic**						
*Methanobrevibacter arboriphilicus*	Hydrogenotrophic	Enzyme assay	N.D.	N.D.	N.D.	Hammel et al., 1984
*Methanosarcina acetivorans* C2A	Aceticlastic	Cultivation/enzyme assay	>150 kPa	Methane, acetate, formate	∼20	Rother and Metcalf, 2004; Oelgeschläger and Rother, 2009
*Methanosarcina barkeri*	Aceticlastic	Cultivation/enzyme assay	>100 kPa	Hydrogen, Methane	∼65	O’Brien et al., 1984; Bott et al., 1986
*Methanobacterium formicicum*	Hydrogenotrophic	Cultivation	N.D.	N.D.	No growth	Kluyver and Schnellen, 1947
*Methanosaeta concillii*	Aceticlastic	Enzyme assay	N.D.	N.D.	No growth	Jetten et al., 1989
**Thermophilic**						
*Methanothermobacter thermoautotrophicus*	Hydrogenotrophic	Cultivation/enzyme assay	50 kPa	Methane, hydrogen	∼200	Daniels et al., 1977; Wasserfallen et al., 2000
*Methanosarcina thermophila*	Aceticlastic	Cultivation	>2 kPa	Hydrogen, Methane	N.D.	Zinder and Anguish, 1992
*Methanothrix* sp. *Strain CALS-1*	Aceticlastic	Cultivation	<2 kPa	Methane	No growth	Zinder and Anguish, 1992
*Archaeoglobus fulgidus^B^*	Sulfate reducer	Cultivation	>136 kPa	Acetate, formate	∼10	Henstra et al., 2007a

*Not determined parameters are marked N.D.*

*^A^Forward arrows (>) indicate inhibitory levels have not been reached; numbers displayed are the maximal level tested. Reverse arrows (<) indicate the tested level was the highest tested and the inhibitory concentration lies below this level.*

*^B^Archaeoglobus fulgidus is not capable of generating methane, but is displayed here due to its capacity to generate acetate and formate from CO, like M. acetivorans.*

**FIGURE 5:**
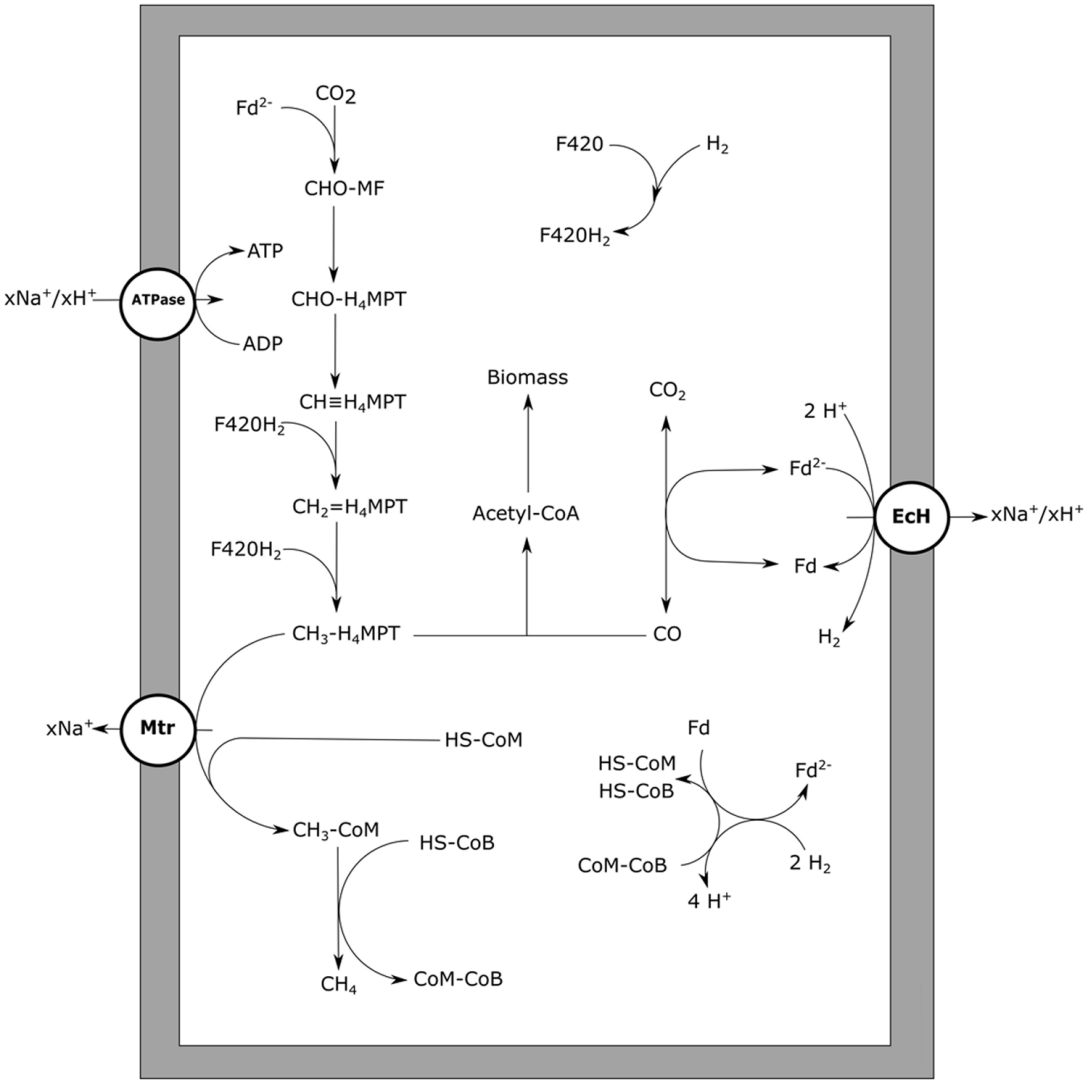
**Carbon monoxide metabolism of hydrogenotrophic methanogens**. CO driven methanogenesis with hydrogen as an intermediate is displayed. xH^+^ or xNa^+^ indicate translocation of an undefined number of protons or sodium ions, respectively. Reactions are not displayed stoichiometrically. EcH, energy converting hydrogenase; Mtr, methyl-H_4_MPT:HS-CoM methyltransferase; ATPase, ATP synthase; Fd, ferredoxin; MF, methanofuran; H_4_MPT, tetrahydromethanopterin; HS-CoM, coenzyme M; HS-CoB, coenzyme B.

In autotrophic, hydrogen-utilizing methanogens, CO is an intermediate of the anabolic reductive acetyl-CoA pathway, a pathway functionally similar to the Wood–Ljungdahl pathway in bacteria (Berg et al., 2010). The archaeal CODH required for growth is a nickel-dependent enzyme, just as observed in bacteria (Hammel et al., 1984). For CO to be utilized as energy source by methanogens, a CODH has to be present that can function in the CO oxidizing direction, and suitable cofactors for electron transfer to the methanogenic metabolism should be available. Thermophilic *Methanothermobacter thermoautotrophicus* is capable of growing on CO, but slowly; at CO pressures up to 50 kPa at a rate of 1% compared to its growth rate on H_2_/CO_2_ (Daniels et al., 1977). The genome of *M. thermoautotrophicus* (Smith et al., 1997) codes for a single CODH enzyme with ACS as a neighboring gene, which are used for anabolism during growth on hydrogen (Stupperich et al., 1983). Growing *M. thermoautotrophicus* on CO as sole electron donor, results in methane formation and small amounts of H_2,_ suggesting hydrogen is an intermediate or side product of the metabolism. In hydrogenotrophic methanogens an EcH is present, which is involved in the reduction of ferredoxin with H_2_, driven by a proton gradient (Thauer et al., 2010). Generation of reduced ferredoxin by CODH allows for the reverse reaction to take place, generating a proton gradient. This additionally results in the formation of hydrogen, which subsequently can be used for reduction of CO_2_ to methane (**Figure 5**). Metabolic activity using CO, with H_2_ as intermediate, is also observed in *Methanosarcina barkeri* (Fischer and Thauer, 1990) and *Methanosarcina thermophila* (Terlesky and Ferry, 1988; Zinder and Anguish, 1992). Experiments with cell extracts and washed cells of *M. thermophila* show increased hydrogenogenic activity upon exposure to CO (Zinder and Anguish, 1992). When growing *M. thermophila* on acetate, hydrogen production was detected besides methane formation, suggesting coupling of CO oxidation to proton reduction (Terlesky and Ferry, 1988). *M. barkeri* also has the capability of oxidizing CO coupled to formation of hydrogen, resulting in the formation of a proton gradient (Bott and Thauer, 1989). Upon addition of methanogenic inhibitors, cell suspensions of *M. barkeri* were still capable of utilizing CO, producing hydrogen and additionally resulting in formation of ATP (Bott et al., 1986). If a hydrogenogenic metabolism can sustain growth in *M. barkeri* is unclear. Without methanogenic inhibitors, the formed hydrogen is further oxidized to form methane, allowing for growth (O’Brien et al., 1984). Judging from the carboxydotrophic generation time of *M. barkeri* (∼65 h) and *M. thermoautotrophicus* (∼200 h), the methanogenic metabolism on CO is not very efficient. For both strains, hydrogen accumulation is observed during methanogenic carboxydotrophic growth (Daniels et al., 1977; O’Brien et al., 1984). This suggests hydrogenases, required for the formation of methane from the intermediately formed hydrogen, are inhibited by CO. The involved hydrogenases, such as the heterodisulfide reductase-associated hydrogenase and the F420-reducing hydrogenase, are of the [Ni–Fe] type (Thauer et al., 2010) and can be expected to be relatively resistant, but not insensitive, to CO (De Lacey et al., 2007). Additionally, *M. thermoautotrophicus* contains an iron-dependent methylenetetrahydromethanopterin dehydrogenase, which is involved in the conversion of methenyltetrahydromethanopterin to methylenetetrahydromethanopterin directly using hydrogen as a donor (Zirngibl et al., 1990). This hydrogenase is found in several hydrogenotrophic methanogens, and is mainly expressed under nickel-deprived conditions (Afting et al., 1998; Afting et al., 2000). The enzyme is susceptible to CO (Lyon et al., 2004) and is therefore a potential target for inhibition during carboxydotrophic growth. Despite the indication that hydrogenases are a limiting factor for carboxydotrophic methanogenesis, inhibition of other enzymes by CO cannot be ruled out as only limited information is available on the mechanisms of CO toxicity in methanogens.

During aceticlastic methanogenesis, CO is an intermediate originating from the splitting of acetyl-CoA by the CODH/ACS complex (Grahame, 2003). Subsequently, CODH is used to further oxidize CO and provide electrons for reduction of the methyl-group to methane. Methanogenic bioreactors fed with acetate accumulated CO to levels up to 0.25 Pa, likely resulting from its role as intermediate in aceticlastic methanogenesis (Hickey and Switzenbaum, 1990). Obligate aceticlastic methanogens such as *Methanothrix* sp. strain *CALS-1*, were observed to accumulate CO to low partial pressures while metabolizing acetate (Zinder and Anguish, 1992). Upon addition of low amounts of CO, it was consumed till equilibrium levels of 0.16 Pa. Addition of CO to levels of 2 kPa caused inhibition of growth on acetate. Obligate aceticlastic methanogens contain a low level of hydrogenases and are in general assumed not to utilize these enzymes for their energy metabolism (Deppenmeier et al., 1996; Smith and Ingram-Smith, 2007). This makes it unlikely that inhibition of hydrogenases results in CO toxicity for these methanogens. However, in contrast to EcH containing methanogens, the electrons released from CO oxidation cannot be coupled to proton reduction, requiring other pathways to re-oxidize formed reduction equivalents. The methyl-branch of the reductive acetyl-CoA pathway could theoretically fulfill this role, as the genes are present in the genome of aceticlastic *Methanosaeta*/*Methanothrix* species (Zhu et al., 2012). All of these organisms, however, lack the ability to grow on H_2_/CO_2_ or formate (Thauer et al., 2008), suggesting no activity of this pathway in the CO_2_-reducing direction. The genes of the methyl-branch coded for in these species are phylogenetically similar to genes present in methylotrophic methanogens, which use this branch in the oxidative direction, in order to generate reduction equivalents for biosynthesis (Zhu et al., 2012). This is further supported by C^13^ labeling studies in *Methanosaeta harundinacea* which confirms activity in the oxidizing but not in the reducing direction (Zhu et al., 2012). This suggests the methyl-branch of the reductive acetyl-CoA pathway in aceticlastic methanogens is not optimal for re-oxidation of reduced cofactors, as is suggested for hydrogenotrophic methanogens (**Figure 5**). When exposed to elevated levels of CO this could result in an over-reduced state of the cell, making it difficult for obligate aceticlastic methanogens to utilize it as a substrate.

**FIGURE 6:**
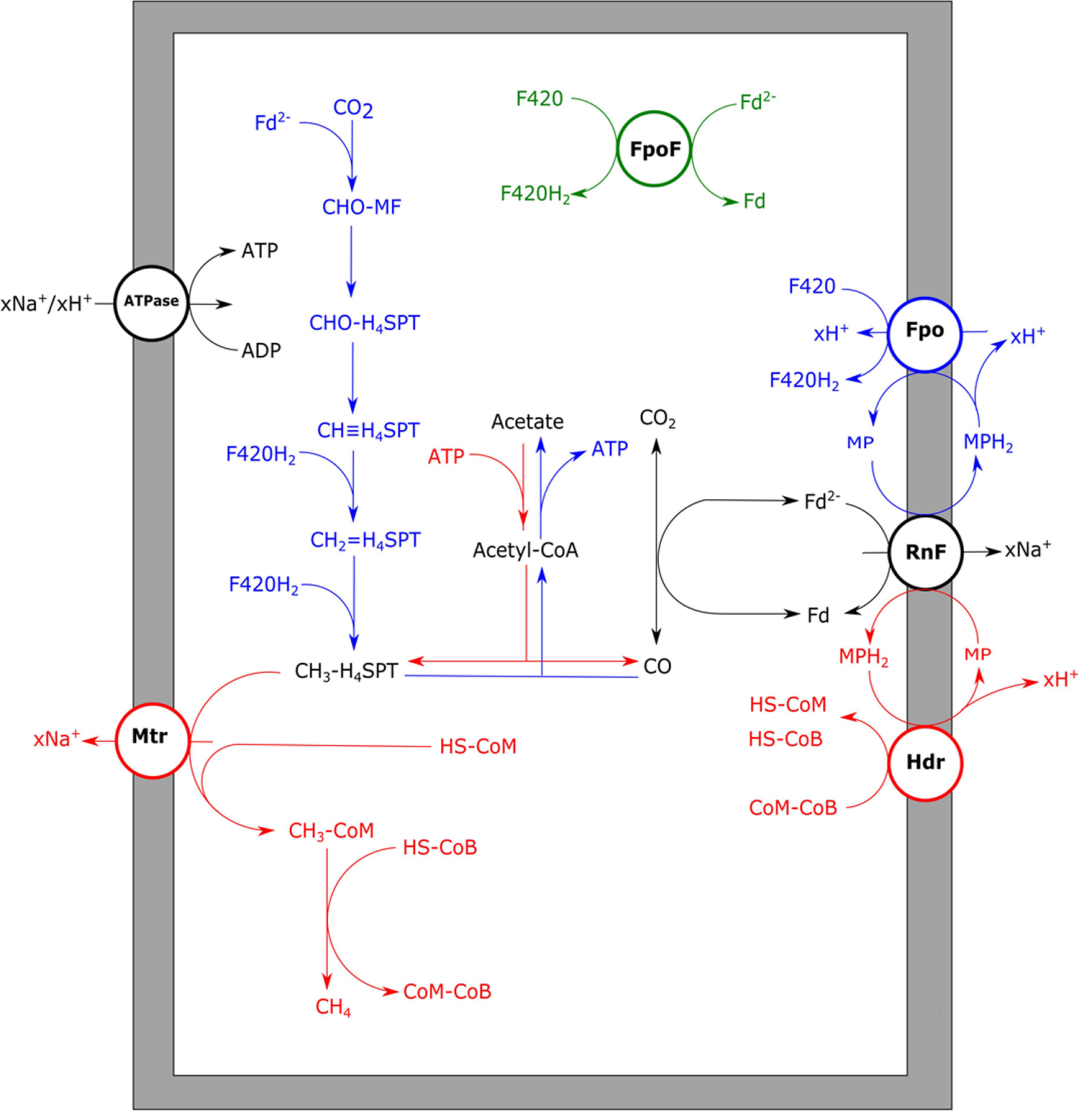
**Aceticlastic and carboxydotrophic metabolism of *Methanosarcina acetivorans***. Aceticlastic methanogenic pathway proposed for *M. acetivorans* (red) and carboxydotrophic pathway proposed for *M. acetivorans* (blue). For carboxydotrophic growth either RnF/Fpo (blue) or FpoF (green) are proposed to generate reduced F420 from ferredoxin. xH^+^ or xNa^+^ indicate translocation of an undefined number of protons or sodium ions, respectively. RnF, RnF-like complex; ATPase, ATP synthase; Mtr, methyl-H_4_SPT:HS-CoM methyltransferase; Fpo, F420 dehydrogenase complex; FpoF, F420 dehydrogenase subunit F; Hdr, heterodisulfide reductase; Fd, ferredoxin; MF, methanofuran; H_4_SPT, tetrahydrosarcinapterin; HS-CoM, coenzyme M; HS-CoB, coenzyme B.

The only methanogen which appears to deal quite well with CO is *Methanosarcina acetivorans*, which was initially isolated from marine sediments (Sowers et al., 1984). In addition to methane, acetate and formate were observed to be the main end products from CO (Rother and Metcalf, 2004). Additionally, *M. acetivorans* was found to produce methylated-thiols from CO (Oelgeschläger and Rother, 2009). The *M. acetivorans* genome codes for two isoforms of CODH/ACS, Cdh1, and Cdh2, which are both considered to be functional in acetyl-CoA anabolism and catabolism (Matschiavelli et al., 2012). The expression levels of the two isoforms are theorized to be regulated on transcriptional and posttranscriptional level by a CdhA subunit (CdhA3), which is suggested to act as a CO sensor (Matschiavelli et al., 2012). In addition to the CODH/ACS complex, two monofunctional CODH, CooS1F, and CooS2, were found to assist in removal of the CO at high CO partial pressures (Rother et al., 2007). For *M. acetivorans* no hydrogen formation is observed during growth on CO, which is supported by the fact that it is devoid of any significant hydrogen metabolism (Sowers et al., 1984). Despite the inability of *M. acetivorans* to grow on H_2_/CO_2_, its genome codes for homologs of the methyl-branch of the reductive acetyl-CoA pathway (Galagan et al., 2002). Proteomic analysis shows that these genes are more abundantly expressed during CO-dependent growth when compared to growth on acetate or methanol (Lessner et al., 2006). This suggests that, in contrast to what is proposed for *Methanosaeta/Methanotrix* species, *M. acetivorans* uses the methyl-branch of the reductive acetyl-CoA pathway to regenerate its reduction equivalents. However, due to its lack of hydrogenases it is unclear how the organism couples oxidation of CO to reduction of F420, which is required to operate this pathway. *M. acetivorans* was shown to express a sodium dependent “RnF-like” complex when metabolizing acetate (Li et al., 2007; Schlegel et al., 2012). It is speculated that this RnF complex couples ferredoxin oxidation to reduction of methanophenazine, subsequently passing on the electrons to the heterodisulfide reductase (Hdr) complex, involved in HS-CoM/HS-CoB regeneration (Li et al., 2007; **Figure 6**). Proteomic data of *M. acetivorans* show that in cells grown on CO the F420-oxidizing:Fpo complex is relatively more abundant, which suggests a role in CO metabolism (Lessner et al., 2006). This protein complex possibly operates in combination with the RnF complex, to catalyze ferredoxin-dependent F420 reduction (Lessner et al., 2006; **Figure 6**). An alternative for coupling ferredoxin oxidation to F420 reduction is via a subunit from the Fpo complex which partly resides in the cytoplasm: FpoF. This subunit was found to catalyze ferredoxin:F420 reduction in EcH knockout mutants of *Methanosarcina mazei*, whereas knockout mutants of the FpoF subunit did not show this activity (Welte and Deppenmeier, 2011). Despite the lack of genes coding for formate dehydrogenases in the genome of *M. activorans* (Galagan et al., 2002), formate is produced in addition to acetate and methane during carboxydotrophic growth. Formate is suggested to originate from activity of formyl-methanofuran dehydrogenase and is theorized to act as a redox exhaust of the cell during CO driven growth (Matschiavelli and Rother, 2015).

*Methanosarcina acetivorans* is not the only archaeon producing acetate and formate from CO, as also the hyperthermophilic, sulfate-reducing *Archaeoglobus fulgidus* generates these products during carboxydotrophic growth in absence of sulfate. In *A. fulgidus*, however, both the RnF and EcH complexes are not coded for in the genome (Hocking et al., 2014). A FpoF homolog, FqoF, is encoded for in the genome of *A. fulgidus* (Brüggemann et al., 2000), which could take part in the coupling of ferredoxin oxidation to F420 reduction. The absence of an EcH complex poses also a question for the redox balance of the organism, as hydrogen cannot be used as a redox exhaust. It is possible that formate is formed in a similar way as theorized for *M. acetivorans*, but it can also be formed via F420-dependent formate dehydrogenases, allowing favorable redox balance during growth.

## Comparison and Application of Fermentative Carboxydotrophic Metabolism

Comparing the three main variants of fermentative CO metabolism, it is obvious that similar sets of enzymes and pathways are used. Hydrogenogenic microorganisms seem to be the most efficient CO-utilizers due to their relatively simple and “redox-closed” energy metabolism. This allows the water–gas shift mechanism to operate at a rate independent of the rest of the metabolism, minimizing metabolic stress. To maximize the energetic yield on CO, a methanogenic or acetogenic microorganism would first have to perform hydrogenogenesis, conserving energy via an EcH complex, subsequently gaining energy from the use of hydrogen for methanogenesis or acetogenesis. Hydrogenotrophic methanogens and acetogens like *M. thermoacetica* seem to make use of this strategy, maximizing the ATP yield per CO utilized. However, despite these optimized energy yields, these organisms have no outstanding growth performance on CO. This is likely related to the CO sensitivity of hydrogenases, essential for this type of metabolism. Acetogens which employ RnF complexes, seem less prone to inhibition by CO, but need to couple CO oxidation stoichiometrically to the Wood–Ljungdahl pathway in order to prevent an over-reduced state of the cell. In relatively fast growing acetogenic bacteria, this is prevented by utilizing alternative pathways to re-oxidize cofactors faster. Loss of “energy-rich” reduction equivalents in these re-oxidizing reactions lowers the overall energy yield of the cell. However, this is expected to allow for better maintenance of redox balance, resulting in reduced stress for the overall metabolism. Still net energy is assumed to be conserved by CO-utilizing acetogens, such as *C. ljungdahlii*, during acetyl-CoA formation. This suggests a large and diverse range of products can be formed from carboxydotrophic metabolism, making it interesting for future research and bio-based applications.

## Author Contributions

MD, Drafted and wrote the manuscript; AS, reviewed and revised the manuscript; DS, reviewed and revised the manuscript. All authors gave approval for publication of the manuscript.

## Conflict of Interest Statement

The authors declare that the research was conducted in the absence of any commercial or financial relationships that could be construed as a potential conflict of interest.
